# CKD Prevalence in the Military Health System: Coded Versus Uncoded CKD

**DOI:** 10.1016/j.xkme.2021.03.015

**Published:** 2021-06-02

**Authors:** Jenna M. Norton, Lindsay Grunwald, Amanda Banaag, Cara Olsen, Andrew S. Narva, Eric Marks, Tracey P. Koehlmoos

**Affiliations:** 1 Department of Preventive Medicine and Biostatistics, Uniformed Services University of the Health Sciences, Bethesda, MD; 2 Henry M Jackson Foundation for the Advancement of Military Medicine, Bethesda, MD; 3 College of Agriculture, Urban Sustainability & Environmental Sciences, University of the District of Columbia, Washington, DC; 4 Division of Nephrology, Department of Medicine, Uniformed Services University of the Health Sciences, Bethesda, MD

**Keywords:** Chronic kidney disease, chronic renal disease, chronic renal insufficiency, electronic health record, medical record, estimated glomerular filtration rate, proteinuria, electronic phenotype

## Abstract

**Rationale & Objective:**

Chronic kidney disease (CKD) is common but often goes unrecorded.

**Study Design:**

Cross-sectional.

**Setting & Participants:**

Military Health System (MHS) beneficiaries aged 18 to 64 years who received care during fiscal years 2016 to 2018.

**Predictors:**

Age, sex, active duty status, race, diabetes, hypertension, and numbers of kidney test results.

**Outcomes:**

We defined CKD by *International Classification of Diseases, Tenth Revision* (*ICD-10*) code and/or a positive result on a validated electronic phenotype that uses estimated glomerular filtration rate and measures of proteinuria with evidence of chronicity. We defined coded CKD by the presence of an *ICD-10* code. We defined uncoded CKD by a positive e-phenotype result without an *ICD-10* code.

**Analytical Approach:**

We compared coded and uncoded populations using 2-tailed *t* tests (continuous variables) and Pearson χ^2^ test for independence (categorical variables).

**Results:**

The MHS population included 3,330,893 beneficiaries. Prevalence of CKD was 3.2%, based on *ICD* code and/or positive e-phenotype result. Of those identified with CKD, 63% were uncoded. Compared with beneficiaries with coded CKD, those with uncoded CKD were younger (aged 45 ± 13 vs 52 ± 11 years), more often women (54.4% vs 37.6%) and active duty (20.2% vs 12.5%), and less often of Black race (18.5% vs 31.5%) or with diabetes (23.5% vs 43.5%) or hypertension (46.6% vs 77.1%; *P* < 0.001). Beneficiaries with coded (vs uncoded) CKD had greater numbers of kidney test results (*P* < 0.001).

**Limitations:**

Use of cross-sectional administrative data prevents inferences about causality. The CKD e-phenotype may fail to capture CKD in individuals without laboratory data and may underestimate CKD.

**Conclusions:**

The prevalence of CKD in the MHS is ~3.2%. Beneficiaries with well-known CKD risk factors, such as older age, male sex, Black race, diabetes, and hypertension, were more likely to be coded, suggesting that clinicians may be missing CKD in groups traditionally considered lower risk, potentially resulting in suboptimal care.

## Graphical abstract

**Figure:**
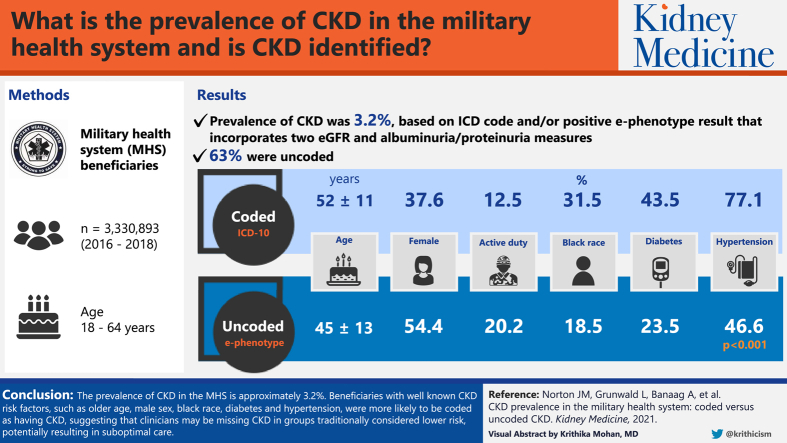

**Plain-Language Summary**

Chronic kidney disease (CKD) often is not recorded with a diagnosis code in the medical record. However, laboratory data can be used to find people with CKD. We used both laboratory and diagnosis code data to identify the total number of people with CKD in the Military Health System (MHS). We compared people with CKD who had a diagnosis code with those who did not have a diagnosis code. We found that 3.2% of the MHS population has CKD. Most (63%) CKD was uncoded. Compared with people with coded CKD, people with uncoded CKD were in groups usually considered lower risk: younger, women, active duty, White race, and without diabetes or hypertension.

More than 30 million American adults (~15% of the US adult population) are estimated to have chronic kidney disease (CKD),^1^ which is characterized by progressive and long-term loss of kidney function that may lead to end-stage kidney disease (ESKD). Individuals with CKD experience substantial morbidity and mortality, including disproportionate rates of hospitalization, cardiovascular disease, mineral and bone disorders, anemia, metabolic acidosis, malnutrition, acute kidney injury, psychiatric illnesses, and reduced quality of life.^2^, ^3^, ^4^, ^5^, ^6^

In addition, CKD imposes a substantial financial burden. In 2016, care for Medicare beneficiaries with recognized CKD or ESKD cost >$114 billion, representing 23% of total Medicare fee-for-service spending despite accounting for only ~13% of the Medicare population.^2^ In ESKD, the disproportionate costs are even more extreme; people with ESKD reflect <1% of the Medicare population but account for 7% of spending.^2^ These significant costs are potentially modifiable because effective strategies exist to slow the progression of CKD and reduce potential complications.^7^

Despite the substantial human and financial costs associated with CKD and the high prevalence of CKD in the general population, little is known about CKD in the nearly 9.5 million beneficiaries of the Military Health System (MHS). Although the active duty population is notably healthier than the general population, only ~20% of MHS beneficiaries are active duty personnel, with the remaining beneficiaries composed of retirees and service members’ families.^8^ Recent analyses of rates of diabetes mellitus and hypertension, the primary causes of ESKD, in the MHS are limited. However, available data suggest that the prevalence of these CKD risk factors may be considerable.^9^^,^^10^

The MHS Data Repository (MDR) contains health data for MHS beneficiaries, including laboratory results for beneficiaries who receive direct care at Military Treatment Centers, making assessment of CKD in the MHS possible. *International Classification of Diseases* (*ICD*) codes are typically inadequate for identifying patients with CKD given low diagnosis rates based solely on *ICD* coding. A systematic review of various studies validating the prevalence of CKD assessed by *ICD* codes against either estimated glomerular filtration rate (eGFR) value or medical record review found that use of *ICD* codes vastly underestimated true CKD prevalence, with sensitivity ranging from 8% to 83%.^11^ A separate systematic review of 19 observational studies that validated diagnostic and procedural codes for CKD found poor sensitivity, with a median of 41% and a range from 3% to 81%.^12^

Because CKD is defined by objective laboratory measures, a laboratory-data–based electronic (e-) phenotype for CKD has the potential to more accurately identify cases of CKD using electronic health record data such as is available through the MDR.^13^ Application of CKD e-phenotypes inclusive of laboratory measures to electronic health record data has demonstrated ability to identify cases of CKD with high accuracy,^14^^,^^15^ outperforming *ICD* codes alone.^15^ A National Institute of Diabetes and Digestive and Kidney Diseases (NIDDK) working group recently developed a CKD e-phenotype based on eGFR and measures of proteinuria, including urinary albumin-creatinine ratio (UACR), urinary protein-creatinine ratio (UPCR), and dipstick urinary albumin, that identified CKD from the electronic health record across 4 health care settings with 99% sensitivity and 99% specificity.^14^

To understand the prevalence of CKD in adult MHS beneficiaries, we used *ICD, Tenth Revision* (*ICD-10*) codes to identify previously diagnosed cases of CKD, as well as the NIDDK CKD e-phenotype to identify probable, but uncoded, cases of CKD. We compared the prevalence of coded and uncoded CKD and explored factors associated with uncoded CKD.

## Methods

### Data Source

This cross-sectional study used data from the MDR under the Comparative Effectiveness and Provider Induced Demand Collaboration (EPIC) project, which has been previously described.^16^ The MDR captures, archives, validates, and merges data for the approximately 9.5 million beneficiaries of the MHS, including all in- and outpatient visits in Department of Defense facilities (direct care) and/or civilian facilities in which the Military’s TRICARE Health Plan was the payer (purchased care). Before data are made available through the MDR, they are thoroughly cleaned, including identification of likely coding errors, assessment for data not missing at random, and imputation of missing values.^17^ For all direct care visits (ie, care provided within military treatment facilities), data include vital signs, body mass index, self-reported tobacco use, medications, and laboratory results, among other variables.^17^ However, data from the civilian fee-for-service (purchased care) environment are limited to the contents of the claim for billing purposes and lack details on outcomes or results of the clinical encounter (eg, laboratory findings). The MDR does not include treatment for service members in combat zones or care administered though Veterans Administration facilities. Further, the MDR has previously been used in studies designed to evaluate epidemiology and quality of health care delivery in a variety of clinical contexts, including surgical care, women’s health, and pediatrics.^18^, ^19^, ^20^

### Study Population

We identified all active duty and retired military personnel and their adult dependents or dependent survivors who received health care through the MHS during the 3-year period from October 1, 2015, to September 30, 2018. Adults aged 18 to 64 years were included in the sample. Adults 65 years and older were excluded because Medicare rather than TRICARE is the primary payer in this population. Inactive guard/reserve, active guard/reserve (if not included as active duty), and dependents of inactive and active guard/reserve were excluded due to the infrequency with which this population accesses TRICARE services.

### Variables of Interest

Coded CKD was defined by *ICD-10* code (Table S1). Any CKD was defined by presence of an *ICD-10* code for CKD and/or laboratory markers of CKD, as defined by the NIDDK CKD e-phenotype.^14^ The e-phenotype defines CKD as 2 consecutive laboratory results indicative of CKD (including eGFR < 60 mL/min/1.73 m^2^, UACR ≥ 30 mg/g, UPCR ≥ 150 mg/g, and/or urine dipstick result ≥ 1+) separated by at least 90 days. This analysis applied the more specific, less-sensitive version of the e-phenotype, wherein the Black race correction factor is used in estimating GFR for individuals of unknown race, dipstick urinary albumin cutoff for CKD is 1+ or greater (rather than trace or greater), and the UPCR cutoff for CKD is ≥150 mg/g (rather than ≥50 mg/g), to err on the side of capturing more severe CKD. Individuals with only a single abnormal laboratory result (ie, no evidence of chronicity) and those missing eGFR, serum creatinine (Scr), UACR, UPCR, and dipstick urinary albumin values (ie, none of the 5 values present) were categorized as negative for phenotyped CKD. Importantly, the NIDDK e-phenotype could not be applied to beneficiaries who receive care through purchased care because laboratory data are not available for this population. Individuals who did not have an *ICD-10* diagnosis code for CKD but were phenotype positive for CKD were categorized as having uncoded CKD (−, +). Subcategories of CKD were also identified for populations who had an *ICD-10* code for CKD and were e-phenotype positive (+, +), had an *ICD-10* code for CKD but had no laboratory data available (+, / ) and had an *ICD-10* code for CKD but were e-phenotype negative (+, −). CKD stage was determined using eGFRs according to Kidney Disease: Improving Global Outcomes criteria.

Dialysis and transplant recipients were identified by the presence of indicative *ICD-10* or *Current Procedural Terminology* codes (Tables S2-S5). Diabetes, hypertension, depression, and HIV-positive status were identified by *ICD-10* code using National Committee for Quality Assurance value sets available from the National Library of Medicine’s Value Set Authority Center (Tables S6-S9).^21^ Body mass index was calculated based on height and weight, excluding biologically implausible values for height and weight (height < 111.8 cm [<44 inches] or >228.6 cm [>90 inches] and weight <24.9 kg [<55 pounds] or >453.6 kg [>1,000 pounds]),^22^ and was categorized as obese (≥30 kg/m^2^), overweight (≥25 and <30 kg/m^2^), or normal/underweight (<25 kg/m^2^).

For each beneficiary, sex, race, birth year, benefits category, marital status, and number of Scr, eGFR, UACR, UPCR, and dipstick urinary albumin measurements were recorded from the MDR. Benefits category was defined as active duty, dependent, retired, or dependent survivor. In addition, for each beneficiary, the sponsor’s military rank, branch of service, and home zip code were captured. Sponsor’s military rank, a commonly used proxy for socioeconomic status,^23^^,^^24^ was defined as Senior Officers (O-5 to O-10), including Warrant Officers (WO-1 to WO-4), Junior Officers (O-1 to O-4), Senior Enlisted (E-5 to E-9), and Junior Enlisted (E-1 to E-4). Branch of service was categorized as Army, Air Force, Marine Corps, Navy, or other.

### Data Analysis

We calculated proportions of the total study population with any CKD, coded CKD, and uncoded CKD. Characteristics of the any CKD, coded CKD, uncoded CKD (−, +), *ICD-10* and phenotype positive (+, +), *ICD-10* positive and phenotype negative (+, −), and *ICD-10* positive with missing laboratory data (+, / ) populations were described using mean with standard deviation and/or median with interquartile range for continuous or discrete variables (age and number of laboratory measurements) and frequency distributions with percentages for categorical variables (race, sex, benefits category, rank, branch of service, body mass index, income, and comorbid conditions). To enable identification of groups at higher risk for having uncoded CKD, unadjusted means and frequency distributions were compared across the coded and uncoded CKD populations using 2-tailed *t* tests and Pearson χ^2^ test for independence, respectively. For each *t* test, the equality of variances assumption was checked and the Satterthwaite method was used when we could not assume equal variances. In addition, sensitivity analyses were run to compare any, coded, and uncoded CKD using an eGFR cutoff of <45 mL/min/1.73 m^2^ for the e-phenotype. *P* ≤ 0.05 was considered statistically significant. Analyses for each variable were based on the observed values only, with missing values excluded from analysis. Analyses were conducted using SAS, version 9.4 (SAS Institute Inc). This study was found exempt by the Uniformed Services University of the Health Sciences Institutional Review Board (Ref #912960). Informed consent was not necessary for this study due to use of deidentified data.

## Results

The total study population consisted of 3,330,893 MHS beneficiaries. We found that 3.2% of the MHS population had CKD identified either by *ICD-10* code or laboratory values indicative of CKD, accounting for 105,504 people. Of these, 38,688 (37%) had an *ICD-10* code for CKD recorded in the MDR, while 66,816 (63%) were uncoded and identified by laboratory values alone. Of note, 53% of the total population but only 2% of the coded CKD population had no kidney test results (Scr, eGFR, UACR, UPCR, or dipstick urinary album) recorded in the MDR. Further, 58% of individuals with coded CKD had kidney test results that did not meet the CKD e-phenotype criteria.

Table 1 shows characteristics of the total, coded, and uncoded CKD populations within the MHS. The total CKD population was 48 years of age on average, 48% women, predominantly of White (47%) or Black (23%) race, and predominantly Senior Enlisted (75%). Of the total CKD population, 39% were retired, 30% were dependents of nonactive duty beneficiaries (eg, retirees and survivors), 17% were active duty, and 14% were dependents of active duty. Both hypertension (57.8%) and diabetes (30.8%) were common in the total CKD population. Approximately half (49%) the CKD population had at least 1 proteinuria measurement (UACR, UPCR, or dipstick urinary albumin) recorded in the MDR, with only 36% having the preferred UACR test. Virtually all (99.2%) had a kidney function test (Scr or eGFR).

**Table 1:** Characteristics of Populations With Any, Coded, and Uncoded CKD in the MHS

	Any CKD	Coded CKD	Uncoded CKD	*P*^a^
Number (%)	105,504 (100%)	38,688 (35%)	66,816 (65%)	
Age, y				
Mean (SD)	48 (12.9)	52 (10.6)	45 (13.5)	<0.001
Median (IQR)	51 (39, 58)	54 (46, 60)	48 (34, 57)	
Female sex	50,867 (48.2%)	14,547 (37.6%)	36,320 (54.4%)	<0.0001
Beneficiary category				
Active duty dependent	14,513 (13.8%)	3,103 (8.0%)	11,410 (17.1%)	<0.001
Retired	41,044 (38.9%)	19,581 (50.6%)	21,463 (32.1%)	
Other dependent	31,616 (30.0%)	11,160 (28.9%)	20,456 (30.6%)	
Active duty	18,331 (17.4%)	4,844 (12.5%)	13,487 (20.2%)	<0.001^b^
Race				
White	49,697 (47.1%)	16,153 (41.8%)	33,544 (50.2%)	<0.001
Black	24,551 (23.3%)	12,197 (31.5%)	12,354 (18.5%)	<0.001^c^
AAPI	4,790 (4.5%)	2,163 (5.6%)	2,627 (3.9%)	
AIAN	372 (0.4%)	111 (0.3%)	261 (0.4%)	
Other	13,171 (12.5%)	4,547 (11.8%)	8,624 (12.9%)	
Unknown	2,996 (2.8%)	735 (1.9%)	2,261 (3.4%)	
Missing	9,927 (9.4%)	2,782 (7.2%)	7,145 (10.7%)	
Rank				
Junior Enlisted	7,952 (7.5%)	1,329 (3.4%)	6,623 (9.9%)	<0.001
Senior Enlisted	79,506 (75.4%)	30,257 (78.2%)	49,249 (73.7%)	
Junior Officer	5,333 (5.1%)	1,727 (4.5%)	3,606 (5.4%)	
Senior Officer	12,712 (12.1%)	5,374 (13.9%)	7,338 (11.0%)	
Other	d	d	0 (0.0%)	
Married	74,393 (70.5%)	28,518 (73.7%)	45,875 (68.7%)	<0.001
Branch of service				
Army	39,988 (37.9%)	15,827 (40.9%)	24,161 (36.2%)	<0.001
Air Force	26,094 (24.7%)	11,881 (30.7%)	14,213 (21.3%)	
Marine Corps	4,317 (4.1%)	1,902 (4.9%)	2,415 (3.6%)	
Navy	33,465 (31.7%)	8,513 (22.0%)	24,952 (37.3%)	
Other	1,640 (1.6%)	565 (1.5%)	1,075 (1.6%)	
Diabetes	32,503 (30.8%)	16,809 (43.5%)	15,694 (23.5%)	<0.001
Hypertension	60,955 (57.8%)	29,836 (77.1%)	31,119 (46.6%)	<0.001
Depression	12,362 (11.7%)	4,831 (12.5%)	7,531 (11.3%)	<0.001
HIV	465 (0.4%)	359 (0.9%)	106 (0.2%)	<0.001
Dialysis	1,772 (1.7%)	1,772 (4.6%)	0.0 (0%)	<0.001
Transplant	1,065 (1.0%)	1,065 (2.8%)	0.0 (0%)	<0.001
BMI (missing = 292,246)				
Obese	51,561 (48.9%)	20,940 (54.1%)	30,621 (45.8%)	<0.001
Overweight	35,689 (33.8%)	12,568 (32.5%)	23,121 (34.6%)	
Normal/under	17,417 (16.5%)	4,828 (12.48%)	12,589 (18.8%)	
Zip code MHI (missing = 427,864)				
Mean (SD)	$60,145 ($19,089)	$65,910 ($22,152)	$56,825 ($16,171)	<0.001
Median (IQR)	$55,251 ($47,737, $67,344)	$60,936 ($50,206, $77,114)	$52,856 ($47,141, $62,747)	
No. of urinary albumin measures^e^	1.1 (1.6)	1.6 (1.9)	0.8 (1.4)	<0.001
No. of UACR measures^e^	0.9 (1.5)	1.3 (1.8)	0.6 (1.2)	<0.001
No. of UPCR measures^e^	0.3 (1.5)	0.8 (2.3)	0.1 (0.5)	<0.001
No. of Scr measures^e^	10.5 (18.3)	16.9 (27.3)	6.9 (8.0)	<0.001
No. of eGFR measures^e^	6.1 (9.5)	9.1 (14.2)	4.3 (4.2)	<0.001
Phenotype positive	82,159 (77.8%)	15,343 (39.6%)	66,816 (100%)	<0.001
Urinary albumin test	47,504 (45.0%)	24,379 (63.0%)	23,125 (34.6%)	<0.001
UACR test	38,391 (36.4%)	20,042 (51.8%)	18,349 (27.5%)	<0.001
UPCR test	12,588 (11.9%)	9,857 (25.5%)	2,731 (4.1%)	<0.001
Any proteinuria test	52,682 (49.9%)	27,507 (71.1%)	25,175 (37.7%)	<0.001
Any kidney test	104,644 (99.2%)	37,828 (97.8%)	66,816 (100%)	<0.001
CKD stage				
G3a	14,056 (13.3%)	8,858 (22.9%)	5,198 (7.8%)	<0.001
G3b	3,938 (3.7%)	3,165 (8.2%)	773 (1.2%)	
G4	1,441 (1.4%)	1,325 (3.4%)	116 (0.2%)	
G5	1,057 (1.0%)	1,024 (2.7%)	33 (0.1%)	

Abbreviations: AAPI, Asian Americans and Pacific Islanders; AIAN, American Indian and Alaska Native; BMI, body mass index; CKD, chronic kidney disease; eGFR, estimated glomerular filtration rate; IQR, interquartile range; MHI, median household income; MHS, Military Health System; Scr, serum creatinine; SD, standard deviation; UACR, urinary albumin-creatinine ratio; UPCR, urinary protein-creatinine ratio.

a *P* values from χ^2^ tests and *t* tests between coded and uncoded CKD.

b Compares active duty with non–active duty.

c Compares Black race with non-Black race.

d Censored due to small cell size.

e Mean (SD) number of tests among those who had any test.

Those with coded CKD were 52 years of age on average, 37.6% women, 41.8% of White race, and 31.5% of Black race, whereas those with uncoded CKD were 45 years of age on average, 54.4% women, 50.2% of White race, and 18.5% of Black race. About half (50.6%) of the coded CKD population was retired and 12.5% were active duty, compared with 32.1% and 20.2%, respectively, of the uncoded CKD population. Among those with coded CKD, 77.1% had hypertension and 43.5% had diabetes, whereas in the uncoded CKD population, only 46.6% had hypertension and 23.5% had diabetes. Further, 71.1% of the coded CKD population had at least 1 proteinuria measurement, whereas only 37.7% of the uncoded CKD population had a proteinuria measurement. Finally, beneficiaries with uncoded CKD had less severe stages of CKD compared with those with coded CKD. However, uncoded CKD spanned CKD 3a-5.

When comparing coded and uncoded CKD populations, individuals with coded CKD were aged 52 years on average, significantly older than those with uncoded CKD, aged 45 years on average (*P* < 0.001). Those with coded compared with uncoded CKD were less likely to be women and active duty but more likely to be of Black race and have diabetes or hypertension (all *P* < 0.001). Among those with test results recorded in the MHS, those with coded CKD had greater numbers of urinary albumin, UACR, UPCR, Scr, and eGFR results (all *P* < 0.001).

Table 2 compares characteristics of populations with coded CKD by e-phenotype category. Of the 38,688 beneficiaries with coded CKD, 15,343 (39.7%) had concordant kidney test results (ie, indicative of CKD) recorded in the MDR, while 22,485 (58.1%) had discordant kidney test results (ie, not indicative of CKD). Only 860 (2%) did not have kidney laboratory results recorded in the MDR. Individuals with discordant codes and laboratory test results were younger, less often women, more often active duty, and less likely to have diabetes and hypertension; had fewer kidney tests performed; and had less severe CKD stage.

**Table 2:** Characteristics of Coded CKD Population (n = 38,688) With e-Phenotype-Positive, e-Phenotype-Negative, and Missing Laboratory Values

	Phenotype Positive	Phenotype Negative	Laboratory Values Missing
No. (%)	15,343 (39.7%)	22,485 (58.1%)	860 (2.2%)
Age, y			
Mean (SD)	54.5 (8.7)	49.6 (11.1)	46.8 (13.6)
Median (IQR)	57 (51, 61)	52 (43, 59)	50 (37, 58)
Female sex (col %)	6,404 (41.7%)	7,767 (34.5%)	376 (43.7%)
Beneficiary category			
Active duty dependent	954 (6.2%)	2,003 (8.9%)	146 (17.0%)
Retired	8,292 (54.0%)	10,971 (48.8%)	318 (37.0%)
Other dependent	5,339 (34.8%)	5,551 (24.7%)	270 (31.4%)
Active duty	758 (4.9%)	3,960 (17.6%)	126 (14.7%)
Race			
White	6,347 (41.4%)	9,492 (42.2%)	314 (36.5%)
Black	4,565 (29.8%)	7,409 (33.0%)	223 (25.9%)
AAPI	1,065 (6.9%)	1,059 (4.7%)	39 (4.5%)
AIAN	34 (0.2%)	74 (0.3%)	a
Other	2,001 (13.0%)	2,461 (11.0%)	85 (9.9%)
Unknown	303 (2.0%)	396 (1.8%)	36 (4.2%)
Missing	1,028 (6.7%)	1,594 (7.1%)	160 (18.6%)
Rank			
Junior Enlisted	333 (2.2%)	937 (4.2%)	59 (6.9%)
Senior Enlisted	12,551 (81.8%)	17,070 (75.9%)	636 (74.0%)
Junior Officer	571 (3.7%)	1,100 (4.9%)	56 (6.5%)
Senior Officer	1,888 (12.3%)	3,378 (15.0%)	108 (12.6%)
Other	0 (0.0%)	0 (0.00%)	a
Married	11,607 (75.7%)	16,421 (73.0%)	490 (57.0%)
Branch of service			
Army	5,652 (36.8%)	9,856 (43.8%)	319 (37.1%)
Air Force	4,585 (29.9%)	7,008 (31.2%)	288 (33.5%)
Marine Corps	712 (4.6%)	1,134 (5.0%)	56 (6.5%)
Navy	4,194 (27.3%)	4,143 (18.4%)	176 (20.5%)
Other	200 (1.3%)	344 (1.5%)	21 (2.4%)
Diabetes	8,887 (57.9%)	7,695 (34.2%)	227 (26.4%)
Hypertension	13,508 (88.0%)	15,910 (70.8%)	418 (48.6%)
Depression	1,953 (12.7%)	2,832 (12.6%)	46 (5.4%)
HIV	122 (0.8%)	234 (1.0%)	a
Dialysis	1,061 (6.9%)	603 (2.7%)	108 (12.6%)
Transplant	504 (3.3%)	474 (2.1%)	87 (10.1%)
BMI (missing = 292,246)			
Obese	8,967 (58.4%)	11,625 (51.7%)	348 (40.5%)
Overweight	4,586 (29.9%)	7,746 (34.5%)	236 (27.4%)
Normal/under	1,737 (11.4%)	2,917 (13.0%)	174 (20.2%)
Zip code MHI (missing = 427,864)			
Mean (SD)	$65,106 ($21,776)	$66,587 ($22,481)	$62,639 ($19,222)
Median (IQR)	$60,753 ($49,871, $75,031)	$61,069 ($50,621, $78,398)	$58,479 ($49,420, $72,284)
No. of urinary albumin measures^b^	2.3 (2.2)	1.2 (1.5)	0
No. of UACR measures^b^	2.0 (2.1)	0.8 (1.3)	0
No. of UPCR measures^b^	1.2 (2.8)	0.5 (1.9)	0
No. of Scr measures^b^	20.4 (29.6)	14.5 (25.3)	0
No. of eGFR measures^b^	11.2 (15.6)	7.8 (13.0)	0
Phenotype positive	15,343 (100%)	0 (0.0%)	0 (0.0%)
Urinary albumin test	11,636 (75.8%)	12,743 (56.7%)	0 (0.0%)
UACR test	10,227 (66.7%)	9,815 (43.7%)	0 (0.0%)
UPCR test	5,334 (34.8%)	4,523 (20.1%)	0 (0.0%)
Any proteinuria	12,845 (83.7%)	14,662 (65.2%)	0 (0.0%)
Any kidney test	15,343 (100%)	22,485 (100%)	0 (0.0%)
CKD stage			
3a	5,921 (38.6%)	2,937 (13.1%)	0 (0.0%)
3b	2,746 (17.9%)	419 (1.9%)	0 (0.0%)
4	1,131 (7.4%)	194 (0.9%)	0 (0.0%)
5	818 (5.3%)	206 (0.9%)	0 (0.0%)

Abbreviations: AAPI, Asian Americans and Pacific Islanders; AIAN, American Indian and Alaska Native; BMI, body mass index; CKD, chronic kidney disease; eGFR, estimated glomerular filtration rate; IQR, interquartile range; MHI, median household income; Scr, serum creatinine; SD, standard deviation; UACR, urinary albumin-creatinine ratio; UPCR, urinary protein-creatinine ratio.

a Censored due to small cell size.

b Mean (SD) number of tests among those who had any test.

Table 3 shows the characteristics of the total, coded, and uncoded CKD populations within the MHS using an eGFR cutoff of <45 mL/min/1.73 m^2^ in the e-phenotype. When narrowing the phenotyped CKD population to more advanced CKD, total CKD prevalence decreases to 39,610 people, or just >1% of the MHS population. Most (98%) of this advanced CKD population had an *ICD-10* code for CKD. Individuals with uncoded CKD were on average older, less often active duty, and more often White and had fewer kidney test results.

**Table 3:** Characteristics of Populations With Any, Coded, and Uncoded CKD in the MHS, With e-Phenotype eGFR Cutoff < 45 mL/min/1.73 m^2^

	Any CKD	Coded CKD	Uncoded CKD	*P*^a^
No. (%)	39,610 (100%)	38,688 (97.7%)	922 (2.3%)	
Age, y				
Mean (SD)	51.6 (10.6)	51.5 (10.6)	58.1 (7.7)	<0.001
Median (IQR)	55 (46,60)	54 (46,60)	61 (56,63)	
Female sex	15,106 (38.14%)	14,547 (37.6%)	559 (60.6%)	<0.001
Beneficiary category				
Active duty dependent	3,144 (7.94%)	3,103 (8.0%)	41 (4.5%)	<0.001
Retired	19,950 (50.4%)	19,581 (50.6%)	369 (40.0%)	
Other dependent	11,640 (29.4%)	11,160 (28.9%)	480 (52.1%)	
Active duty	4,876 (12.3%)	4,844 (12.5%)	32 (3.5%)	<0.001^b^
Race				
White	16,684 (42.1%)	16,153 (41.8%)	531 (57.6%)	<0.001
Black	12,351 (31.2%)	12,197 (31.5%)	154 (16.7%)	<0.001^c^
AAPI	2,207 (5.6%)	2,163 (5.6%)	44 (4.8%)	
AIAN	114 (0.3%)	111 (0.3%)	∗∗	
Other	4,664 (11.8%)	4,547 (11.8%)	117 (12.7%)	
Unknown	762 (1.9%)	735 (1.9%)	27 (2.9%)	
Missing	2,828 (7.1%)	2,782 (7.2%)	46 (5.0%)	
Rank				
Junior Enlisted	1,355 (3.4%)	1,329 (3.4%)	26 (2.8%)	0.07
Senior Enlisted	31,015 (78.3%)	30,257 (78.2%)	758 (82.2%)	
Junior Officer	1,760 (4.4%)	1,727 (4.5%)	33 (3.6%)	
Senior Officer	5,479 (13.8%)	5,374 (13.9%)	105 (11.4%)	
Other	d	d	0 (0.0%)	
Married	29,233 (73.8%)	28,518 (73.7%)	715 (77.6%)	0.009
Branch of service				
Army	16,235 (41.0%)	15,827 (40.9%)	408 (44.3%)	0.21
Air Force	12,151 (30.7%)	11,881 (30.7%)	270 (29.3%)	
Marine Corps	1,952 (4.9%)	1,902 (4.9%)	50 (5.4%)	
Navy	8,694 (22.0%)	8,513 (22.0%)	181 (19.6%)	
Other	578 (1.5%)	565 (1.5%)	13 (1.4%)	
Diabetes	17,186 (43.4%)	16,809 (43.5%)	377 (40.9%)	0.12
Hypertension	30,443 (76.9%)	29,836 (77.1%)	607 (65.8%)	<0.001
Depression	4,946 (12.5%)	4,831 (12.5%)	115 (12.5%)	0.99
HIV	364 (0.9%)	359 (0.9%)	d	0.23
Dialysis N	1,772 (4.5%)	1,772 (4.6%)	0 (0.0%)	<0.001
Transplant	1,065 (2.7%)	1,065 (2.8%)	0 (0.0%)	<0.001
BMI (missing = 292,246)				
Obese	21,430 (54.1%)	20,940 (54.1%)	490 (53.2%)	<0.001
Overweight	12,833 (32.4%)	12,568 (32.5%)	265 (8.7%)	
Normal/under	4,950 (12.5%)	4,828 (12.48%)	122 (13.2%)	
Zip code MHI (missing = 427,864)				
Mean (SD)	$65,804 ($22,099)	$65,910 ($22,152)	$61,365 ($19,247)	<0.001
Median (IQR)	$60,936 ($49,990, $76,923)	$60,936 ($50,206, $77,114)	$57,356 ($47,979, $72,284)	
No. of urinary albumin measures^e^	1.6 (1.9)	1.6 (1.9)	1.3 (1.7)	<0.001
No. of UACR measures^e^	1.3 (1.7)	1.3 (1.8)	0.9 (1.5)	<0.001
No. of UPCR measures^e^	0.8 (2.3)	0.8 (2.3)	0.4 (1.5)	<0.001
No. of Scr measures^e^	16.9 (27.3)	16.9 (27.3)	16.8 (25.5)	0.95
No. of eGFR measures^e^	9.1 (14.2)	9.1 (14.2)	9.2 (13.0)	0.87
Phenotype positive	5,617 (14.2%)	4,695 (12.1%)	922 (100%)	<0.001
Urinary albumin test result	24,866 (62.8%)	24,373 (63.0%)	487 (52.8%)	<0.001
UACR test result	20,440 (51.6%)	20,042 (51.8%)	398 (43.2%)	<0.001
UPCR test result	10,005 (25.3%)	9,857 (25.5%)	148 (16.1%)	<0.001
Any proteinuria	28,050 (70.8%)	27,507 (71.1%)	543 (58.9%)	<0.001
Any kidney test	38,750 (97.8%)	37,828 (97.8%)	922 (100%)	<0.001
CKD stage				
3a	8,858 (22.4%)	8,858 (22.9%)	0 (0.0%)	<0.001
3b	3,938 (9.9%)	3,165 (8.2%)	773 (83.8%)	
4	1,441 (3.6%)	1,325 (3.4%)	116 (12.6%)	
5	1,057 (2.7%)	1,024 (2.7%)	33 (3.6%)	

Abbreviations: AAPI, Asian Americans and Pacific Islanders; AIAN, American Indian and Alaska Native; BMI, body mass index; CKD, chronic kidney disease; eGFR, estimated glomerular filtration rate; IQR, interquartile range; MHI, median household income; MHS, Military Health System; Scr, serum creatinine; SD, standard deviation; UACR, urinary albumin-creatinine ratio; UPCR, urinary protein-creatinine ratio.

a *P* values from χ^2^ tests and *t* tests between coded and uncoded CKD.

b Compares active duty with non–active duty.

c Compares Black beneficiaries to white beneficiaries.

d Censored due to small cell size.

e Mean (SD) number of tests among those who had any test.

## Discussion

To date, few published data are available on the burden of CKD in the MHS. This analysis suggests that 3.2% of the MHS population—105,504 MHS beneficiaries—may have CKD, based on data from federal fiscal years 2016 through 2018. Prior estimates of CKD prevalence in the MHS by Oliver et al^25^^,^^26^ have been lower. An analysis using diagnosis codes in the full TRICARE population estimated the 2015 prevalence of CKD at between 2.6% and 2.9%.^25^ A separate study in the subpopulation of MHS beneficiaries who receive exclusive direct care from Military Treatment Centers estimated the 2015 prevalence of CKD at 2.5%, based on the presence of at least 2 abnormal laboratory values indicative of CKD (ie, eGFR, UACR, or UPCR) separated by 90 or more days.^26^ Given the incomplete overlap of diagnosed and phenotyped CKD, the higher prevalence identified in this analysis likely results from the combined use of diagnosis codes and/or laboratory values to capture CKD. In addition, the NIDDK e-phenotype for CKD used in this analysis includes dipstick urinary albumin as a measure of proteinuria, with CKD indicated in individuals with 2 or more results of ≥1+ separated by at least 90 days, whereas the prior analysis by Oliver et al did not use dipstick urinary albumin to identify CKD. As in other health care settings,^14^ dipstick urinary albumin is more commonly measured than other measures of proteinuria in the MHS and therefore addition of this laboratory result likely increased sensitivity for identifying CKD.

Importantly, the 3.2% prevalence of CKD found in this analysis likely underestimates the true prevalence of CKD in the MHS population. Diagnosis codes have been demonstrated to undercapture cases of CKD.^11^^,^^26^ Although use of the NIDDK e-phenotype to capture probable cases of CKD by laboratory values will increase the sensitivity of CKD detection, the less sensitive more specific versions of the NIDDK e-phenotype were used in the analysis and therefore the phenotype may have failed to capture some cases of CKD. In addition, kidney test results must be available to apply the e-phenotype. However, 53% of the included MHS population did not have any Scr, eGFR, UACR, UPCR, or dipstick urinary albumin results recorded in the MDR on which to apply the e-phenotype. This may, to a large degree, result from the lack of laboratory data in the MDR for any purchased care interactions received from both network and non-network TRICARE-authorized civilian health care professionals, institutions, pharmacies, and suppliers. Just more than half (~54%) of TRICARE expenditures are for purchased care services.^8^ Therefore, phenotype-positive laboratory results may exist for MHS beneficiaries who were tested through purchased care interactions, which we were unable to include in this analysis.

The large proportion (58.1%) of coded CKD beneficiaries who had kidney test results not indicative of CKD recorded in the MDR may reflect some combination of individuals coded with early stages of disease (eg, G1A1 and G2A1), errant codes for CKD, or missingness of more recent laboratory data indicative of CKD. Because MHS beneficiaries may move between direct and purchased care, it is possible that available laboratory data do not reflect a given beneficiary’s most recent data.

Only 37% of the probable CKD population had a diagnosis code for CKD, notably higher than the rate of coded CKD among laboratory-identified CKD in the Medicare population identified recently by Diamantidis et al.^27^ Uncoded CKD was more common in early stages of CKD and in groups traditionally considered to be of lower risk for progressive CKD: younger adults, females, people of White race, and those without diabetes or hypertension, which is similar to patterns seen in the Medicare population.^2^^,^^27^ The younger age in the uncoded CKD population may reflect the comparatively less severe stage of CKD given that the association between uncoded CKD and age reverses when the e-phenotype is limited to more advanced CKD. However, females and White beneficiaries remain less likely to be coded when restricting the e-phenotype to more advanced CKD. These findings are largely consistent with studies conducted in primary care practices in the United Kingdom, which found that CKD was more frequently coded in men, individuals of older age, and those with relevant comorbid conditions.^28^^,^^29^

Contrary to our findings in the MHS, the studied UK primary care practices had higher rates of uncoded CKD in practices with predominantly minority patients.^29^ However, differences in demographic, social, and contextual factors between the UK primary care population and the MHS population must be acknowledged. The high rates of CKD coding in Black MHS beneficiaries is perhaps unsurprising, given that many Black-White health care disparities that persist in the United States are absent in the MHS,^30^, ^31^, ^32^, ^33^ perhaps due to the universal health care coverage provided through the MHS, the high rate of employment for MHS beneficiaries (or their sponsors), and/or differences in clinical cultures and practices.

Lack of CKD coding in these traditionally low CKD risk groups suggests that clinicians may be missing CKD diagnoses despite available laboratory data indicative of CKD. As a result, these individuals with uncoded CKD may not be receiving appropriate management to slow the progression of the disease and address potential complications. Prior research has shown associations between lack of clinical coding for CKD and guideline-discordant care.^28^ In addition, the presence of coded CKD is associated with a greater likelihood of patient awareness of the CKD diagnosis.^34^ As expected, beneficiaries with uncoded CKD had fewer numbers of urinary albumin, UACR, UPCR, Scr, and eGFR results, suggesting that kidney function and damage are not monitored as closely in this patient population. Application of the NIDDK CKD e-phenotype^14^ in population health management initiatives in the clinical setting could potentially help identify these individuals likely to have CKD, thereby enabling improved disease management.

This analysis is a novel first attempt to identify all cases of CKD in the MHS population using both *ICD-10* codes and laboratory values indicative of CKD. Additional strengths include the large sample size and application of a validated laboratory value−based e-phenotype to improve the sensitivity of CKD detection.

However, important limitations must be acknowledged. Data used in this analysis are administrative and thus are intended for use in claims adjudication and not research. Due to the cross-sectional nature of the data, causality cannot be inferred. More than half the total MHS population lacked kidney test results on which to apply the CKD e-phenotype. As a result, the CKD e-phenotype may fail to capture CKD in individuals who have laboratory values indicative of CKD acquired through purchased care. The phenotype also cannot be applied to any individual who has simply not received any kidney tests. As a result, we may underestimate the true burden of CKD in the MHS.

This novel study for the first time identified the prevalence of CKD in the MHS at ~3.2%. Of MHS beneficiaries with probable CKD, 63% lacked an *ICD*-*10* code for CKD, suggesting that they may not be receiving appropriate management to slow progression and address complications. Beneficiaries with well-known risk factors for CKD (eg, older age, male sex, Black race, diagnosed diabetes, and diagnosed hypertension) were more likely to have a CKD *ICD-10* code, suggesting that clinicians may be missing CKD in groups traditionally considered lower risk despite available laboratory data to assess CKD status.
