# Supplementary material for: CKD Prevalence in the Military Health System: Coded Versus Uncoded CKD
Source: Kidney Med. 2021 Jun 2;3(4):586–595.e1. doi: 10.1016/j.xkme.2021.03.015 (PMC8350811; doi:10.1016/j.xkme.2021.03.015)
Supplement: Supplementary File (PDF) — Tables S1-S9. [file mmc1.pdf]

Supplemental Materials

Table of Contents

Supplemental Table 1: ICD-10 Codes to Identify CKD from the MDR

Supplemental Table 2: CPT Codes to Identify Dialysis Recipients from the MDR

Supplemental Table 3: ICD-10 Codes to Identify Dialysis Recipients from the MDR

Supplemental Table 4: CPT Codes to Identify Transplant Recipients from the MDR

Supplemental Table 5: ICD-10 Codes to Identify Transplant Recipients from the MDR

Supplemental Table 6: ICD-10 Codes to Identify Diagnosed Diabetes from the MDR

Supplemental Table 7: ICD-10 Codes to Identify Diagnosed Hypertension from the MDR

Supplemental Table 8: ICD-10 Codes to Identify Diagnosed Depression from the MDR

Supplemental Table 9: ICD-10 Codes to Identify Diagnosed HIV from the MDR

Table S1: ICD-10 Codes to Identify CKD from the MDR

Code(s)	Description
N18.1, N18.2, N18.3, N18.4, N18.5, N18.6, N18.9	Chronic kidney disease
Q61.2, Q61.3	Polycystic kidney disease
N01.3, N08, N03.0, N03.1, N03.2, N03.3, N03.4, N03.5, N03.6, N03.7, N03.8, N03.9	Glomerulonephritis/nephritis/nephrotic syndrome
E08.22, E09.22, E10.21, E10.22, E10.29, E11.21, E11.22, E11.29, E13.22	Diabetic nephropathy
I12.0, I12.9, I13.0, I13.1, I13.2, I13.9	Hypertensive nephrosclerosis

Table S2: CPT Codes to Identify Dialysis Recipients from the MDR (1)

Code	Description
3066F	Documentation of treatment for nephropathy
36800, 36810, 36815	Insertion of cannula for hemodialysis, other purpose…
36818 - 36820	Arteriovenous anastomosis, open…
36821, 36831	Thrombectomy, open, arteriovenous fistula…
36832, 36833	Revision, open, arteriovenous fistula…
90935, 90937	Hemodialysis procedure with single evaluation…
90940	Hemodialysis access flow study to determine blood flow…
90945, 90947	Dialysis procedure other than hemodialysis…
90951 - 90962	ESRD related services monthly…
90963 - 90966	ESRD related services for home dialysis per full month…
90967 - 90970	ESRD related services for dialysis less than a full month…
90989, 90993	Dialysis training, patient, including helper…
90997	Hemoperfusion
90999, 99512	Unlisted dialysis procedure, inpatient or outpatient…
G0257	Unscheduled or emergency dialysis treatment for an ESRD…
G9231	Documentation of ESRD, dialysis, renal transplant…
S2065	Simultaneous pancreas kidney transplantation…
S9339	Home therapy; peritoneal dialysis, administrative…
36145	Introduction of needle or intracatheter; arteriovenous shunt created for dialysis…
36147	Introduction of needle and/or catheter, arteriovenous shunt created for dialysis…
90918 - 90921	ESRD related services per full month…
90925	ESRD related services (less than full month)…
G0308 – G0319	ESRD related services during the course of treatment…
G0320 – G0323	ESRD related services for home dialysis patients per full month…
G0324 – G0327	ESRD related services for home dialysis (less than full month)…
G0392, G0393	Transluminal balloon angioplasty, percutaneous; for maintenance of hemodialysis access…

Table S3: ICD-10 Codes to Identify Dialysis Recipients from the MDR (1)

Code	Description
N18.6	End stage renal disease
Z49	Encounter for care involving renal dialysis
Z91.15	Patient's noncompliance with renal dialysis
Z99.2	Dependence on renal dialysis

Table S4: CPT Codes to Identify Transplant Recipients from the MDR (1)

Code	Description
00868	Anesthesia for extraperitoneal procedures in lower abdomen, including urinary tract; renal transplant (recipient) (units: 10)
50340	Recipient nephrectomy (separate procedure)
50360, 50365	Renal allotransplantation; implantation of graft…
50380	Renal autotransplantation, reimplantation of kidney

Table S5: ICD-10 Codes to Identify Transplant Recipients from the MDR

Code	Description
Z94.0	Kidney transplant status

Table S6: ICD-10 Codes to Identify Diagnosed Diabetes from the MDR

Code	Description
E10.10	Type 1 diabetes mellitus with ketoacidosis without coma
E10.11	Type 1 diabetes mellitus with ketoacidosis with coma
E10.21	Type 1 diabetes mellitus with diabetic nephropathy
E10.22	Type 1 diabetes mellitus with diabetic chronic kidney disease
E10.29	Type 1 diabetes mellitus with other diabetic kidney complication
E10.311	Type 1 diabetes mellitus with unspecified diabetic retinopathy with macular edema
E10.319	Type 1 diabetes mellitus with unspecified diabetic retinopathy without macular edema
E10.321	Type 1 diabetes mellitus with mild nonproliferative diabetic retinopathy with macular edema
E10.3211	Type 1 diabetes mellitus with mild nonproliferative diabetic retinopathy with macular edema, right eye
E10.3212	Type 1 diabetes mellitus with mild nonproliferative diabetic retinopathy with macular edema, left eye
E10.3213	Type 1 diabetes mellitus with mild nonproliferative diabetic retinopathy with macular edema, bilateral
E10.3219	Type 1 diabetes mellitus with mild nonproliferative diabetic retinopathy with macular edema, unspecified eye
E10.329	Type 1 diabetes mellitus with mild nonproliferative diabetic retinopathy without macular edema
E10.3291	Type 1 diabetes mellitus with mild nonproliferative diabetic retinopathy without macular edema, right eye
E10.3292	Type 1 diabetes mellitus with mild nonproliferative diabetic retinopathy without macular edema, left eye
E10.3293	Type 1 diabetes mellitus with mild nonproliferative diabetic retinopathy without macular edema, bilateral
E10.3299	Type 1 diabetes mellitus with mild nonproliferative diabetic retinopathy without macular edema, unspecified eye
E10.331	Type 1 diabetes mellitus with moderate nonproliferative diabetic retinopathy with macular edema
E10.3311	Type 1 diabetes mellitus with moderate nonproliferative diabetic retinopathy with macular edema, right eye
E10.3312	Type 1 diabetes mellitus with moderate nonproliferative diabetic retinopathy with macular edema, left eye
E10.3313	Type 1 diabetes mellitus with moderate nonproliferative diabetic retinopathy with macular edema, bilateral
E10.3319	Type 1 diabetes mellitus with moderate nonproliferative diabetic retinopathy with macular edema, unspecified eye
E10.339	Type 1 diabetes mellitus with moderate nonproliferative diabetic retinopathy without macular edema
E10.3391	Type 1 diabetes mellitus with moderate nonproliferative diabetic retinopathy without macular edema, right eye
E10.3392	Type 1 diabetes mellitus with moderate nonproliferative diabetic retinopathy without macular edema, left eye

E10.3393	Type 1 diabetes mellitus with moderate nonproliferative diabetic retinopathy without macular edema, bilateral
E10.3399	Type 1 diabetes mellitus with moderate nonproliferative diabetic retinopathy without macular edema, unspecified eye
E10.341	Type 1 diabetes mellitus with severe nonproliferative diabetic retinopathy with macular edema
E10.3411	Type 1 diabetes mellitus with severe nonproliferative diabetic retinopathy with macular edema, right eye
E10.3412	Type 1 diabetes mellitus with severe nonproliferative diabetic retinopathy with macular edema, left eye
E10.3413	Type 1 diabetes mellitus with severe nonproliferative diabetic retinopathy with macular edema, bilateral
E10.3419	Type 1 diabetes mellitus with severe nonproliferative diabetic retinopathy with macular edema, unspecified eye
E10.349	Type 1 diabetes mellitus with severe nonproliferative diabetic retinopathy without macular edema
E10.3491	Type 1 diabetes mellitus with severe nonproliferative diabetic retinopathy without macular edema, right eye
E10.3492	Type 1 diabetes mellitus with severe nonproliferative diabetic retinopathy without macular edema, left eye
E10.3493	Type 1 diabetes mellitus with severe nonproliferative diabetic retinopathy without macular edema, bilateral
E10.3499	Type 1 diabetes mellitus with severe nonproliferative diabetic retinopathy without macular edema, unspecified eye
E10.351	Type 1 diabetes mellitus with proliferative diabetic retinopathy with macular edema
E10.3511	Type 1 diabetes mellitus with proliferative diabetic retinopathy with macular edema, right eye
E10.3512	Type 1 diabetes mellitus with proliferative diabetic retinopathy with macular edema, left eye
E10.3513	Type 1 diabetes mellitus with proliferative diabetic retinopathy with macular edema, bilateral
E10.3519	Type 1 diabetes mellitus with proliferative diabetic retinopathy with macular edema, unspecified eye
E10.3521	Type 1 diabetes mellitus with proliferative diabetic retinopathy with traction retinal detachment involving the macula, right eye
E10.3522	Type 1 diabetes mellitus with proliferative diabetic retinopathy with traction retinal detachment involving the macula, left eye
E10.3523	Type 1 diabetes mellitus with proliferative diabetic retinopathy with traction retinal detachment involving the macula, bilateral
E10.3529	Type 1 diabetes mellitus with proliferative diabetic retinopathy with traction retinal detachment involving the macula, unspecified eye
E10.3531	Type 1 diabetes mellitus with proliferative diabetic retinopathy with traction retinal detachment not involving the macula, right eye
E10.3532	Type 1 diabetes mellitus with proliferative diabetic retinopathy with traction retinal detachment not involving the macula, left eye
E10.3533	Type 1 diabetes mellitus with proliferative diabetic retinopathy with traction retinal detachment not involving the macula, bilateral
E10.3539	Type 1 diabetes mellitus with proliferative diabetic retinopathy with traction retinal detachment not involving the macula, unspecified eye

E10.3541	Type 1 diabetes mellitus with proliferative diabetic retinopathy with combined traction retinal detachment and rhegmatogenous retinal detachment, right eye
E10.3542	Type 1 diabetes mellitus with proliferative diabetic retinopathy with combined traction retinal detachment and rhegmatogenous retinal detachment, left eye
E10.3543	Type 1 diabetes mellitus with proliferative diabetic retinopathy with combined traction retinal detachment and rhegmatogenous retinal detachment, bilateral
E10.3549	Type 1 diabetes mellitus with proliferative diabetic retinopathy with combined traction retinal detachment and rhegmatogenous retinal detachment, unspecified eye
E10.3551	Type 1 diabetes mellitus with stable proliferative diabetic retinopathy, right eye
E10.3552	Type 1 diabetes mellitus with stable proliferative diabetic retinopathy, left eye
E10.3553	Type 1 diabetes mellitus with stable proliferative diabetic retinopathy, bilateral
E10.3559	Type 1 diabetes mellitus with stable proliferative diabetic retinopathy, unspecified eye
E10.359	Type 1 diabetes mellitus with proliferative diabetic retinopathy without macular edema
E10.3591	Type 1 diabetes mellitus with proliferative diabetic retinopathy without macular edema, right eye
E10.3592	Type 1 diabetes mellitus with proliferative diabetic retinopathy without macular edema, left eye
E10.3593	Type 1 diabetes mellitus with proliferative diabetic retinopathy without macular edema, bilateral
E10.3599	Type 1 diabetes mellitus with proliferative diabetic retinopathy without macular edema, unspecified eye
E10.36	Type 1 diabetes mellitus with diabetic cataract
E10.37X1	Type 1 diabetes mellitus with diabetic macular edema, resolved following treatment, right eye
E10.37X2	Type 1 diabetes mellitus with diabetic macular edema, resolved following treatment, left eye
E10.37X3	Type 1 diabetes mellitus with diabetic macular edema, resolved following treatment, bilateral
E10.37X9	Type 1 diabetes mellitus with diabetic macular edema, resolved following treatment, unspecified eye
E10.39	Type 1 diabetes mellitus with other diabetic ophthalmic complication
E10.40	Type 1 diabetes mellitus with diabetic neuropathy, unspecified
E10.41	Type 1 diabetes mellitus with diabetic mononeuropathy
E10.42	Type 1 diabetes mellitus with diabetic polyneuropathy
E10.43	Type 1 diabetes mellitus with diabetic autonomic (poly)neuropathy
E10.44	Type 1 diabetes mellitus with diabetic amyotrophy
E10.49	Type 1 diabetes mellitus with other diabetic neurological complication
E10.51	Type 1 diabetes mellitus with diabetic peripheral angiopathy without gangrene

E10.52	Type 1 diabetes mellitus with diabetic peripheral angiopathy with gangrene
E10.59	Type 1 diabetes mellitus with other circulatory complications
E10.610	Type 1 diabetes mellitus with diabetic neuropathic arthropathy
E10.618	Type 1 diabetes mellitus with other diabetic arthropathy
E10.620	Type 1 diabetes mellitus with diabetic dermatitis
E10.621	Type 1 diabetes mellitus with foot ulcer
E10.622	Type 1 diabetes mellitus with other skin ulcer
E10.628	Type 1 diabetes mellitus with other skin complications
E10.630	Type 1 diabetes mellitus with periodontal disease
E10.638	Type 1 diabetes mellitus with other oral complications
E10.641	Type 1 diabetes mellitus with hypoglycemia with coma
E10.649	Type 1 diabetes mellitus with hypoglycemia without coma
E10.65	Type 1 diabetes mellitus with hyperglycemia
E10.69	Type 1 diabetes mellitus with other specified complication
E10.8	Type 1 diabetes mellitus with unspecified complications
E10.9	Type 1 diabetes mellitus without complications
E11.00	Type 2 diabetes mellitus with hyperosmolarity without nonketotic hyperglycemic-hyperosmolar coma (NKHHC)
E11.01	Type 2 diabetes mellitus with hyperosmolarity with coma
E11.10	Type 2 diabetes mellitus with ketoacidosis without coma
E11.11	Type 2 diabetes mellitus with ketoacidosis with coma
E11.21	Type 2 diabetes mellitus with diabetic nephropathy
E11.22	Type 2 diabetes mellitus with diabetic chronic kidney disease
E11.29	Type 2 diabetes mellitus with other diabetic kidney complication
E11.311	Type 2 diabetes mellitus with unspecified diabetic retinopathy with macular edema
E11.319	Type 2 diabetes mellitus with unspecified diabetic retinopathy without macular edema
E11.321	Type 2 diabetes mellitus with mild nonproliferative diabetic retinopathy with macular edema
E11.3211	Type 2 diabetes mellitus with mild nonproliferative diabetic retinopathy with macular edema, right eye
E11.3212	Type 2 diabetes mellitus with mild nonproliferative diabetic retinopathy with macular edema, left eye
E11.3213	Type 2 diabetes mellitus with mild nonproliferative diabetic retinopathy with macular edema, bilateral
E11.3219	Type 2 diabetes mellitus with mild nonproliferative diabetic retinopathy with macular edema, unspecified eye
E11.329	Type 2 diabetes mellitus with mild nonproliferative diabetic retinopathy without macular edema
E11.3291	Type 2 diabetes mellitus with mild nonproliferative diabetic retinopathy without macular edema, right eye

E11.3292	Type 2 diabetes mellitus with mild nonproliferative diabetic retinopathy without macular edema, left eye
E11.3293	Type 2 diabetes mellitus with mild nonproliferative diabetic retinopathy without macular edema, bilateral
E11.3299	Type 2 diabetes mellitus with mild nonproliferative diabetic retinopathy without macular edema, unspecified eye
E11.331	Type 2 diabetes mellitus with moderate nonproliferative diabetic retinopathy with macular edema
E11.3311	Type 2 diabetes mellitus with moderate nonproliferative diabetic retinopathy with macular edema, right eye
E11.3312	Type 2 diabetes mellitus with moderate nonproliferative diabetic retinopathy with macular edema, left eye
E11.3313	Type 2 diabetes mellitus with moderate nonproliferative diabetic retinopathy with macular edema, bilateral
E11.3319	Type 2 diabetes mellitus with moderate nonproliferative diabetic retinopathy with macular edema, unspecified eye
E11.339	Type 2 diabetes mellitus with moderate nonproliferative diabetic retinopathy without macular edema
E11.3391	Type 2 diabetes mellitus with moderate nonproliferative diabetic retinopathy without macular edema, right eye
E11.3392	Type 2 diabetes mellitus with moderate nonproliferative diabetic retinopathy without macular edema, left eye
E11.3393	Type 2 diabetes mellitus with moderate nonproliferative diabetic retinopathy without macular edema, bilateral
E11.3399	Type 2 diabetes mellitus with moderate nonproliferative diabetic retinopathy without macular edema, unspecified eye
E11.341	Type 2 diabetes mellitus with severe nonproliferative diabetic retinopathy with macular edema
E11.3411	Type 2 diabetes mellitus with severe nonproliferative diabetic retinopathy with macular edema, right eye
E11.3412	Type 2 diabetes mellitus with severe nonproliferative diabetic retinopathy with macular edema, left eye
E11.3413	Type 2 diabetes mellitus with severe nonproliferative diabetic retinopathy with macular edema, bilateral
E11.3419	Type 2 diabetes mellitus with severe nonproliferative diabetic retinopathy with macular edema, unspecified eye
E11.349	Type 2 diabetes mellitus with severe nonproliferative diabetic retinopathy without macular edema
E11.3491	Type 2 diabetes mellitus with severe nonproliferative diabetic retinopathy without macular edema, right eye

E11.3492	Type 2 diabetes mellitus with severe nonproliferative diabetic retinopathy without macular edema, left eye
E11.3493	Type 2 diabetes mellitus with severe nonproliferative diabetic retinopathy without macular edema, bilateral
E11.3499	Type 2 diabetes mellitus with severe nonproliferative diabetic retinopathy without macular edema, unspecified eye
E11.351	Type 2 diabetes mellitus with proliferative diabetic retinopathy with macular edema
E11.3511	Type 2 diabetes mellitus with proliferative diabetic retinopathy with macular edema, right eye
E11.3512	Type 2 diabetes mellitus with proliferative diabetic retinopathy with macular edema, left eye
E11.3513	Type 2 diabetes mellitus with proliferative diabetic retinopathy with macular edema, bilateral
E11.3519	Type 2 diabetes mellitus with proliferative diabetic retinopathy with macular edema, unspecified eye
E11.3521	Type 2 diabetes mellitus with proliferative diabetic retinopathy with traction retinal detachment involving the macula, right eye
E11.3522	Type 2 diabetes mellitus with proliferative diabetic retinopathy with traction retinal detachment involving the macula, left eye
E11.3523	Type 2 diabetes mellitus with proliferative diabetic retinopathy with traction retinal detachment involving the macula, bilateral
E11.3529	Type 2 diabetes mellitus with proliferative diabetic retinopathy with traction retinal detachment involving the macula, unspecified eye
E11.3531	Type 2 diabetes mellitus with proliferative diabetic retinopathy with traction retinal detachment not involving the macula, right eye
E11.3532	Type 2 diabetes mellitus with proliferative diabetic retinopathy with traction retinal detachment not involving the macula, left eye
E11.3533	Type 2 diabetes mellitus with proliferative diabetic retinopathy with traction retinal detachment not involving the macula, bilateral
E11.3539	Type 2 diabetes mellitus with proliferative diabetic retinopathy with traction retinal detachment not involving the macula, unspecified eye
E11.3541	Type 2 diabetes mellitus with proliferative diabetic retinopathy with combined traction retinal detachment and rhegmatogenous retinal detachment, right eye
E11.3542	Type 2 diabetes mellitus with proliferative diabetic retinopathy with combined traction retinal detachment and rhegmatogenous retinal detachment, left eye
E11.3543	Type 2 diabetes mellitus with proliferative diabetic retinopathy with combined traction retinal detachment and rhegmatogenous retinal detachment, bilateral
E11.3549	Type 2 diabetes mellitus with proliferative diabetic retinopathy with combined traction retinal detachment and rhegmatogenous retinal detachment, unspecified eye

E11.3551	Type 2 diabetes mellitus with stable proliferative diabetic retinopathy, right eye
E11.3552	Type 2 diabetes mellitus with stable proliferative diabetic retinopathy, left eye
E11.3553	Type 2 diabetes mellitus with stable proliferative diabetic retinopathy, bilateral
E11.3559	Type 2 diabetes mellitus with stable proliferative diabetic retinopathy, unspecified eye
E11.359	Type 2 diabetes mellitus with proliferative diabetic retinopathy without macular edema
E11.3591	Type 2 diabetes mellitus with proliferative diabetic retinopathy without macular edema, right eye
E11.3592	Type 2 diabetes mellitus with proliferative diabetic retinopathy without macular edema, left eye
E11.3593	Type 2 diabetes mellitus with proliferative diabetic retinopathy without macular edema, bilateral
E11.3599	Type 2 diabetes mellitus with proliferative diabetic retinopathy without macular edema, unspecified eye
E11.36	Type 2 diabetes mellitus with diabetic cataract
E11.37X1	Type 2 diabetes mellitus with diabetic macular edema, resolved following treatment, right eye
E11.37X2	Type 2 diabetes mellitus with diabetic macular edema, resolved following treatment, left eye
E11.37X3	Type 2 diabetes mellitus with diabetic macular edema, resolved following treatment, bilateral
E11.37X9	Type 2 diabetes mellitus with diabetic macular edema, resolved following treatment, unspecified eye
E11.39	Type 2 diabetes mellitus with other diabetic ophthalmic complication
E11.40	Type 2 diabetes mellitus with diabetic neuropathy, unspecified
E11.41	Type 2 diabetes mellitus with diabetic mononeuropathy
E11.42	Type 2 diabetes mellitus with diabetic polyneuropathy
E11.43	Type 2 diabetes mellitus with diabetic autonomic (poly)neuropathy
E11.44	Type 2 diabetes mellitus with diabetic amyotrophy
E11.49	Type 2 diabetes mellitus with other diabetic neurological complication
E11.51	Type 2 diabetes mellitus with diabetic peripheral angiopathy without gangrene
E11.52	Type 2 diabetes mellitus with diabetic peripheral angiopathy with gangrene
E11.59	Type 2 diabetes mellitus with other circulatory complications
E11.610	Type 2 diabetes mellitus with diabetic neuropathic arthropathy
E11.618	Type 2 diabetes mellitus with other diabetic arthropathy
E11.620	Type 2 diabetes mellitus with diabetic dermatitis
E11.621	Type 2 diabetes mellitus with foot ulcer
E11.622	Type 2 diabetes mellitus with other skin ulcer
E11.628	Type 2 diabetes mellitus with other skin complications

E11.630	Type 2 diabetes mellitus with periodontal disease
E11.638	Type 2 diabetes mellitus with other oral complications
E11.641	Type 2 diabetes mellitus with hypoglycemia with coma
E11.649	Type 2 diabetes mellitus with hypoglycemia without coma
E11.65	Type 2 diabetes mellitus with hyperglycemia
E11.69	Type 2 diabetes mellitus with other specified complication
E11.8	Type 2 diabetes mellitus with unspecified complications
E11.9	Type 2 diabetes mellitus without complications
E13.00	Other specified diabetes mellitus with hyperosmolarity without nonketotic hyperglycemic-hyperosmolar coma (NKHHC)
E13.01	Other specified diabetes mellitus with hyperosmolarity with coma
E13.10	Other specified diabetes mellitus with ketoacidosis without coma
E13.11	Other specified diabetes mellitus with ketoacidosis with coma
E13.21	Other specified diabetes mellitus with diabetic nephropathy
E13.22	Other specified diabetes mellitus with diabetic chronic kidney disease
E13.29	Other specified diabetes mellitus with other diabetic kidney complication
E13.311	Other specified diabetes mellitus with unspecified diabetic retinopathy with macular edema
E13.319	Other specified diabetes mellitus with unspecified diabetic retinopathy without macular edema
E13.321	Other specified diabetes mellitus with mild nonproliferative diabetic retinopathy with macular edema
E13.3211	Other specified diabetes mellitus with mild nonproliferative diabetic retinopathy with macular edema, right eye
E13.3212	Other specified diabetes mellitus with mild nonproliferative diabetic retinopathy with macular edema, left eye
E13.3213	Other specified diabetes mellitus with mild nonproliferative diabetic retinopathy with macular edema, bilateral
E13.3219	Other specified diabetes mellitus with mild nonproliferative diabetic retinopathy with macular edema, unspecified eye
E13.329	Other specified diabetes mellitus with mild nonproliferative diabetic retinopathy without macular edema
E13.3291	Other specified diabetes mellitus with mild nonproliferative diabetic retinopathy without macular edema, right eye
E13.3292	Other specified diabetes mellitus with mild nonproliferative diabetic retinopathy without macular edema, left eye
E13.3293	Other specified diabetes mellitus with mild nonproliferative diabetic retinopathy without macular edema, bilateral
E13.3299	Other specified diabetes mellitus with mild nonproliferative diabetic retinopathy without macular edema, unspecified eye

E13.331	Other specified diabetes mellitus with moderate nonproliferative diabetic retinopathy with macular edema
E13.3311	Other specified diabetes mellitus with moderate nonproliferative diabetic retinopathy with macular edema, right eye
E13.3312	Other specified diabetes mellitus with moderate nonproliferative diabetic retinopathy with macular edema, left eye
E13.3313	Other specified diabetes mellitus with moderate nonproliferative diabetic retinopathy with macular edema, bilateral
E13.3319	Other specified diabetes mellitus with moderate nonproliferative diabetic retinopathy with macular edema, unspecified eye
E13.339	Other specified diabetes mellitus with moderate nonproliferative diabetic retinopathy without macular edema
E13.3391	Other specified diabetes mellitus with moderate nonproliferative diabetic retinopathy without macular edema, right eye
E13.3392	Other specified diabetes mellitus with moderate nonproliferative diabetic retinopathy without macular edema, left eye
E13.3393	Other specified diabetes mellitus with moderate nonproliferative diabetic retinopathy without macular edema, bilateral
E13.3399	Other specified diabetes mellitus with moderate nonproliferative diabetic retinopathy without macular edema, unspecified eye
E13.341	Other specified diabetes mellitus with severe nonproliferative diabetic retinopathy with macular edema
E13.3411	Other specified diabetes mellitus with severe nonproliferative diabetic retinopathy with macular edema, right eye
E13.3412	Other specified diabetes mellitus with severe nonproliferative diabetic retinopathy with macular edema, left eye
E13.3413	Other specified diabetes mellitus with severe nonproliferative diabetic retinopathy with macular edema, bilateral
E13.3419	Other specified diabetes mellitus with severe nonproliferative diabetic retinopathy with macular edema, unspecified eye
E13.349	Other specified diabetes mellitus with severe nonproliferative diabetic retinopathy without macular edema
E13.3491	Other specified diabetes mellitus with severe nonproliferative diabetic retinopathy without macular edema, right eye
E13.3492	Other specified diabetes mellitus with severe nonproliferative diabetic retinopathy without macular edema, left eye
E13.3493	Other specified diabetes mellitus with severe nonproliferative diabetic retinopathy without macular edema, bilateral

E13.3499	Other specified diabetes mellitus with severe nonproliferative diabetic retinopathy without macular edema, unspecified eye
E13.351	Other specified diabetes mellitus with proliferative diabetic retinopathy with macular edema
E13.3511	Other specified diabetes mellitus with proliferative diabetic retinopathy with macular edema, right eye
E13.3512	Other specified diabetes mellitus with proliferative diabetic retinopathy with macular edema, left eye
E13.3513	Other specified diabetes mellitus with proliferative diabetic retinopathy with macular edema, bilateral
E13.3519	Other specified diabetes mellitus with proliferative diabetic retinopathy with macular edema, unspecified eye
E13.3521	Other specified diabetes mellitus with proliferative diabetic retinopathy with traction retinal detachment involving the macula, right eye
E13.3522	Other specified diabetes mellitus with proliferative diabetic retinopathy with traction retinal detachment involving the macula, left eye
E13.3523	Other specified diabetes mellitus with proliferative diabetic retinopathy with traction retinal detachment involving the macula, bilateral
E13.3529	Other specified diabetes mellitus with proliferative diabetic retinopathy with traction retinal detachment involving the macula, unspecified eye
E13.3531	Other specified diabetes mellitus with proliferative diabetic retinopathy with traction retinal detachment not involving the macula, right eye
E13.3532	Other specified diabetes mellitus with proliferative diabetic retinopathy with traction retinal detachment not involving the macula, left eye
E13.3533	Other specified diabetes mellitus with proliferative diabetic retinopathy with traction retinal detachment not involving the macula, bilateral
E13.3539	Other specified diabetes mellitus with proliferative diabetic retinopathy with traction retinal detachment not involving the macula, unspecified eye
E13.3541	Other specified diabetes mellitus with proliferative diabetic retinopathy with combined traction retinal detachment and rhegmatogenous retinal detachment, right eye
E13.3542	Other specified diabetes mellitus with proliferative diabetic retinopathy with combined traction retinal detachment and rhegmatogenous retinal detachment, left eye
E13.3543	Other specified diabetes mellitus with proliferative diabetic retinopathy with combined traction retinal detachment and rhegmatogenous retinal detachment, bilateral
E13.3549	Other specified diabetes mellitus with proliferative diabetic retinopathy with combined traction retinal detachment and rhegmatogenous retinal detachment, unspecified eye
E13.3551	Other specified diabetes mellitus with stable proliferative diabetic retinopathy, right eye
E13.3552	Other specified diabetes mellitus with stable proliferative diabetic retinopathy, left eye

E13.3553	Other specified diabetes mellitus with stable proliferative diabetic retinopathy, bilateral
E13.3559	Other specified diabetes mellitus with stable proliferative diabetic retinopathy, unspecified eye
E13.359	Other specified diabetes mellitus with proliferative diabetic retinopathy without macular edema
E13.3591	Other specified diabetes mellitus with proliferative diabetic retinopathy without macular edema, right eye
E13.3592	Other specified diabetes mellitus with proliferative diabetic retinopathy without macular edema, left eye
E13.3593	Other specified diabetes mellitus with proliferative diabetic retinopathy without macular edema, bilateral
E13.3599	Other specified diabetes mellitus with proliferative diabetic retinopathy without macular edema, unspecified eye
E13.36	Other specified diabetes mellitus with diabetic cataract
E13.37X1	Other specified diabetes mellitus with diabetic macular edema, resolved following treatment, right eye
E13.37X2	Other specified diabetes mellitus with diabetic macular edema, resolved following treatment, left eye
E13.37X3	Other specified diabetes mellitus with diabetic macular edema, resolved following treatment, bilateral
E13.37X9	Other specified diabetes mellitus with diabetic macular edema, resolved following treatment, unspecified eye
E13.39	Other specified diabetes mellitus with other diabetic ophthalmic complication
E13.40	Other specified diabetes mellitus with diabetic neuropathy, unspecified
E13.41	Other specified diabetes mellitus with diabetic mononeuropathy
E13.42	Other specified diabetes mellitus with diabetic polyneuropathy
E13.43	Other specified diabetes mellitus with diabetic autonomic (poly)neuropathy
E13.44	Other specified diabetes mellitus with diabetic amyotrophy
E13.49	Other specified diabetes mellitus with other diabetic neurological complication
E13.51	Other specified diabetes mellitus with diabetic peripheral angiopathy without gangrene
E13.52	Other specified diabetes mellitus with diabetic peripheral angiopathy with gangrene
E13.59	Other specified diabetes mellitus with other circulatory complications
E13.610	Other specified diabetes mellitus with diabetic neuropathic arthropathy
E13.618	Other specified diabetes mellitus with other diabetic arthropathy
E13.620	Other specified diabetes mellitus with diabetic dermatitis
E13.621	Other specified diabetes mellitus with foot ulcer
E13.622	Other specified diabetes mellitus with other skin ulcer
E13.628	Other specified diabetes mellitus with other skin complications

E13.630	Other specified diabetes mellitus with periodontal disease
E13.638	Other specified diabetes mellitus with other oral complications
E13.641	Other specified diabetes mellitus with hypoglycemia with coma
E13.649	Other specified diabetes mellitus with hypoglycemia without coma
E13.65	Other specified diabetes mellitus with hyperglycemia
E13.69	Other specified diabetes mellitus with other specified complication
E13.8	Other specified diabetes mellitus with unspecified complications
E13.9	Other specified diabetes mellitus without complications
O24.011	Pre-existing type 1 diabetes mellitus, in pregnancy, first trimester
O24.012	Pre-existing type 1 diabetes mellitus, in pregnancy, second trimester
O24.013	Pre-existing type 1 diabetes mellitus, in pregnancy, third trimester
O24.019	Pre-existing type 1 diabetes mellitus, in pregnancy, unspecified trimester
O24.02	Pre-existing type 1 diabetes mellitus, in childbirth
O24.03	Pre-existing type 1 diabetes mellitus, in the puerperium
O24.111	Pre-existing type 2 diabetes mellitus, in pregnancy, first trimester
O24.112	Pre-existing type 2 diabetes mellitus, in pregnancy, second trimester
O24.113	Pre-existing type 2 diabetes mellitus, in pregnancy, third trimester
O24.119	Pre-existing type 2 diabetes mellitus, in pregnancy, unspecified trimester
O24.12	Pre-existing type 2 diabetes mellitus, in childbirth
O24.13	Pre-existing type 2 diabetes mellitus, in the puerperium
O24.311	Unspecified pre-existing diabetes mellitus in pregnancy, first trimester
O24.312	Unspecified pre-existing diabetes mellitus in pregnancy, second trimester
O24.313	Unspecified pre-existing diabetes mellitus in pregnancy, third trimester
O24.319	Unspecified pre-existing diabetes mellitus in pregnancy, unspecified trimester
O24.32	Unspecified pre-existing diabetes mellitus in childbirth
O24.33	Unspecified pre-existing diabetes mellitus in the puerperium
O24.811	Other pre-existing diabetes mellitus in pregnancy, first trimester
O24.812	Other pre-existing diabetes mellitus in pregnancy, second trimester
O24.813	Other pre-existing diabetes mellitus in pregnancy, third trimester
O24.819	Other pre-existing diabetes mellitus in pregnancy, unspecified trimester
O24.82	Other pre-existing diabetes mellitus in childbirth
O24.83	Other pre-existing diabetes mellitus in the puerperium

Table S7: ICD-10 Codes to Identify Diagnosed Hypertension from the MDR

Code	Description
I10	Essential (primary) hypertension
I11.0	Hypertensive heart disease with heart failure
I11.9	Hypertensive heart disease without heart failure
I12.0	Hypertensive chronic kidney disease with stage 5 chronic kidney disease or end stage renal disease
I12.9	Hypertensive chronic kidney disease with stage 1 through stage 4 chronic kidney disease, or unspecified chronic kidney disease
I13.0	Hypertensive heart and chronic kidney disease with heart failure and stage 1 through stage 4 chronic kidney disease, or unspecified chronic kidney disease
I13.10	Hypertensive heart and chronic kidney disease without heart failure, with stage 1 through stage 4 chronic kidney disease, or unspecified chronic kidney disease
I13.11	Hypertensive heart and chronic kidney disease without heart failure, with stage 5 chronic kidney disease, or end stage renal disease
I13.2	Hypertensive heart and chronic kidney disease with heart failure and with stage 5 chronic kidney disease, or end stage renal disease
I15.0	Renovascular hypertension
I15.1	Hypertension secondary to other renal disorders
I15.2	Hypertension secondary to endocrine disorders
I15.8	Other secondary hypertension
I15.9	Secondary hypertension, unspecified

Table S8: ICD-10 Codes to Identify Diagnosed Major Depression from the MDR

Code	Description
F32.0	Major depressive disorder, single episode, mild
F32.1	Major depressive disorder, single episode, moderate
F32.2	Major depressive disorder, single episode, severe without psychotic features
F32.3	Major depressive disorder, single episode, severe with psychotic features
F32.4	Major depressive disorder, single episode, in partial remission
F32.9	Major depressive disorder, single episode, unspecified
F33.0	Major depressive disorder, recurrent, mild
F33.1	Major depressive disorder, recurrent, moderate
F33.2	Major depressive disorder, recurrent severe without psychotic features
F33.3	Major depressive disorder, recurrent, severe with psychotic symptoms
F33.41	Major depressive disorder, recurrent, in partial remission
F33.9	Major depressive disorder, recurrent, unspecified

Table S9: ICD-10 Codes to Identify Diagnosed HIV from the MDR

Code	Description
B20	Human immunodeficiency virus [HIV] disease
B97.35	Human immunodeficiency virus, type 2 [HIV 2] as the cause of diseases classified elsewhere
Z21	Asymptomatic human immunodeficiency virus [HIV] infection status

References

1. Norton JM, Ali K, Jurkovitz CT, Kiryluk K, Park M, Kawamoto K, Shang N, Navaneethan SD, Narva AS, Drawz P: Development and Validation of a Pragmatic Electronic Phenotype for CKD. *Clinical journal of the American Society of Nephrology : CJASN,* 14**:** 1306-1314, 2019
